# Elevating NagZ Improves Resistance to β-Lactam Antibiotics via Promoting AmpC β-Lactamase in *Enterobacter cloacae*

**DOI:** 10.3389/fmicb.2020.586729

**Published:** 2020-11-04

**Authors:** Xianggui Yang, Jun Zeng, Qin Zhou, Xuejing Yu, Yuanxiu Zhong, Fuying Wang, Hongfei Du, Fang Nie, Xueli Pang, Dan Wang, Yingzi Fan, Tingting Bai, Ying Xu

**Affiliations:** ^1^Department of Laboratory Medicine, Clinical Medical College and The First Affiliated Hospital of Chengdu Medical College, Chengdu, China; ^2^Division of Pulmonary and Critical Care Medicine, Clinical Medical College and The First Affiliated Hospital of Chengdu Medical College, Chengdu, China; ^3^Department of Cardiothoracic Surgery, University of Utah, Salt Lake City, UT, United States; ^4^Department of Biotechnology, Chengdu Medical College, Chengdu, China

**Keywords:** *nagZ*, β-lactam antibiotics, resistance, AmpC, *Enterobacter cloacae*

## Abstract

*Enterobacter cloacae* complex (ECC), one of the most common opportunistic pathogens causing multiple infections in human, is resistant to β-lactam antibiotics mainly due to its highly expressed chromosomal AmpC β-lactamase. It seems that regulation of chromosomal AmpC β-lactamase is associated with peptidoglycan recycling. However, underlying mechanisms are still poorly understood. In this study, we confirmed that NagZ, a glycoside hydrolase participating in peptidoglycan recycling in Gram-negative bacteria, plays a crucial role in developing resistance of *E. cloacae* (EC) to β-lactam antibiotics by promoting expression of chromosomal AmpC β-lactamase. Our data shows that NagZ was significantly up-regulated in resistant EC (resistant to at least one type of the third or fourth generation cephalosporins) compared to susceptible EC (susceptible to all types of the third and fourth generation cephalosporins). Similarly, the expression and β-lactamase activity of *ampC* were markedly enhanced in resistant EC. Moreover, ectopic expression of *nagZ* enhanced *ampC* expression and resistance to β-lactam antibiotics in susceptible EC. To further understand functions of NagZ in β-lactam resistance, *nagZ*-knockout EC model (Δ*nagZ* EC) was constructed by homologous recombination. Conversely, *ampC* mRNA and protein levels were down-regulated, and resistance to β-lactam antibiotics was attenuated in Δ*nagZ* EC, while specific complementation of *nagZ* was able to rescue *ampC* expression and resistance in Δ*nagZ* EC. More interestingly, NagZ and its hydrolyzates 1,6-anhydromuropeptides (anhMurNAc) could induce the expression of other target genes of AmpR (a global transcriptional factor), which suggested that the promotion of AmpC by NagZ is mediated AmpR activated by anhMurNAc in EC. In conclusion, these findings provide new elements for a better understanding of resistance in EC, which is crucial for the identification of novel potential drug targets.

## Introduction

*Enterobacter cloacae* complex (ECC), including *E. cloacae* (EC), *Enterobacter asburiae*, *Enterobacter hormaechei*, *Enterobacter kobei*, *Enterobacter ludwigii*, and *Enterobacter nimipressuralis* (Guerin et al., 2015), are widely distributed in nature. They are parts of commensal microbiota in human gastrointestinal tract as well. Over past few decades, ECC has emerged as troublesome pathogens for nosocomial infection worldwide, with an infection rate ranging from 5 to 10% in intensive care unit (ICU) (Mezzatesta et al., 2012; Annavajhala et al., 2019). Among ECC species, *E. cloacae* (EC) is the most significant and frequently isolated in clinical practice, accounting for a high proportion of infections, including 5% of hospital-acquired sepsis, 5% of hospital-acquired pneumonia, 4% of hospital-acquired urinary tract infection, and 10% of postoperative peritonitis (Nicolas et al., 1987; da Silva et al., 2018). The clinical significance of EC has been widely reported especially in the recent 15 years since it has a strong ability to acquire antibiotic resistance, making it the most worrisome microorganism in current era of antibiotics (Mezzatesta et al., 2012).

It is well known that EC has an intrinsic ability to be resistant to ampicillin, amoxicillin/clavulanate, the first and second generation cephalosporins due to its low expression of chromosomal *ampC* gene which encodes AmpC β-lactamase under a basal condition (Jacoby, 2009; Ito et al., 2019). AmpC β-lactamase is the first-discovered bacterial β-lactamase to hydrolyze penicillin in *Escherichia coli* in 1940, but it is not named until 1965 (Eriksson-Grennberg et al., 1965; Eriksson-Grennberg, 1968; Abraham and Chain, 1988). The sequence of AmpC β-lactamase is quite different from penicillin-typed β-lactamase (such as TEM-1), but it has a same amino acid of serine at its active site (Pimenta et al., 2014). For classification, AmpC β-lactamase is classified to be class C based on Ambler method, while it is assigned to be group 1 according to Bush functional classification (Silveira et al., 2018; Mack et al., 2019). The chromosomal AmpC β-lactamase is highly inducible in presence of some β-lactams, such as imipenem, cefoxitin, and clavulanate (Jacoby, 2009; Gomez-Simmonds et al., 2018), but it is still not clear about underlying genetic regulation in AmpC β-lactamase associated with peptidoglycan recycling in *E. cloacae* clinical isolates.

NagZ, a cytosolic glucosaminidase involved in peptidoglycan recycling, has an ability to hydrolyze *N*-acetylglucosaminyl-1,6-anhydromuropeptides (peptidoglycan monomers) to be *N*-acetylglucosaminyl (GlcNAc) and 1,6-anhydromuropeptides (anhMurNAc). anhMurNAc acts as an activated ligand for AmpR in *Pseudomonas aeruginosa* (Stubbs et al., 2008; Huang et al., 2015b). It has been reported that inactivation of NagZ can prevent and revert β-lactam resistance in *P. aeruginosa* (Asgarali et al., 2009; Zamorano et al., 2010b; Acebron et al., 2017), *Y. enterocolitica* (Liu et al., 2017), and *Stenotrophomonas maltophilia* (Huang et al., 2012, 2015a). In addition, NagZ has a moonlighting activity to modulate biofilm accumulation in *Neisseria gonorrhoeae* (Bhoopalan et al., 2016). Despite those promising findings, precise regulation of NagZ to resistance remains largely unknown in EC.

The aims of this study were to determine roles of NagZ in EC resistance development and in chromosomal AmpC β-lactamase regulation. Our study showed that NagZ was overexpressed in resistant EC (resistant to at least one type of the third or fourth generation cephalosporins) compared with susceptible EC (susceptible to all types of the third and fourth generation cephalosporins), complementation of NagZ enhanced EC resistance by up-regulating expression of AmpC. Moreover, NagZ hydrolyzates 1,6-anhydromuropeptides (anhMurNAc) induce the expression of target genes of AmpR. Our findings demonstrated NagZ plays an indispensable role in developing resistance in EC and provided a novel insight into understanding of molecular mechanisms of resistance to β-lactam antibiotics.

## Materials and Methods

### Bacterial Strains, Plasmids, Primers

Detailed information of bacterial strains (Supplementary Table 2), plasmids (Supplementary Table 3), and primers (Supplementary Table 4) used in this study are listed in Supplementary Material.

### Ethics Approval and Consent to Participate

The microorganism research and animal subject research (for preparation of anti-NagZ antibody) were approved by the Ethics Committee of the Clinical Medical College and the First Affiliated Hospital of Chengdu Medical College. After clearly explaining the nature and purposes of this scientific research to all participants, sufficient time was provided for questions and answers, written consents were acquired from all participants.

### Antibiotic Susceptibility Test

Antibiotic susceptibility test was performed by using broth microdilution and Kirby-Bauer method according to protocols recommended by Clinical Laboratory Standard Institute (CLSI, 2018). *E. cloacae* subsp. *cloacae* ATCC 13047 and *E. coli* ATCC 25922 were used for quality control. All antibiotics and culture medium used in antibiotic susceptibility test were purchased from Wenzhou Kangtai company (Bio-kont Co., Ltd., Wenzhou, China). Each assay was performed independently at least three times.

### Generation of Anti-NagZ Antibody

Anti-NagZ antibody was generated through rabbit immunization by an “antigen intersection” strategy immunization and purification (Arora et al., 2014; Zhou et al., 2016). Briefly, *nagZ* coding sequence (CDS) from EC was obtained by polymerase chain reaction (PCR), cloned into a pET28a vector with a 6His-label. Then, pET28a-*nagZ*-6His vector was transformed into *E. coli B21* for expression of NagZ recombinant protein, which was purified by Ni-NAT and identified by electrophoresis. Next, purified NagZ-6His recombinant protein was used to immunize rabbit. Enzyme linked immunosorbent assay (ELISA) was applied to evaluate titer of antiserum (over 1:8000) after immunization of NagZ-6His. Finally, antiserum was purified by affinity of antibody to NagZ-6His-coupled antigen. Western blot showed an excellent specificity of the antibody (Supplementary Figure 1). Reagents and materials used in generation of anti-NagZ antibody were purchased Shenggong Biological Company (Sangon Biotech Co., Ltd., Shanghai, China), primers for obtaining *nagZ* CDS are listed in Supplementary Table 4.

### AmpC β-Lactamase Activity Assay

AmpC β-lactamase activity was determined by a nitrocefin hydrolysis assay as previously described (Cavallari et al., 2013; Guerin et al., 2015). EC isolates were inoculated into LB medium and incubated at 37°C with 250 rpm overnight. It was sub-cultured in LB medium with a concentration of 1:100. When absorbance of OD600 reached 0.8, bacteria were collected and washed once with 1 ml of phosphate buffer (pH 7.0), and resuspended in 1 ml of protein lysate (Sangon Biotech Co., Ltd., Shanghai, China). Samples were placed on ice and lysed by sonication with a microprobe by using a 10-s pulse three times with a 10-s interval during each pulse. The samples were centrifuged at 10,000*g* for 10 min and supernatant was collected. The concentration of protein in supernatant was determined by a protein quantitative kit (Beyotime, Biotechnology, Shanghai, China). The nitrocefin hydrolysis assay was performed in 250 μl of phosphate buffer (pH 7.0) containing 5 μg of total protein and 50 μg/ml nitrocefin (Sigma-Aldrich; Merck KGaA, St. Louis, MO, United States). The hydrolysis rate of nitrocefin was determined at 486 nm at room temperature every 5 min. AmpC β-lactamase activity was calculated by extinction coefficient of nitrocefin 20, 500 M^–1^ cm^–1^, each assay was performed independently at least three times.

### RNA Extraction

The total RNA was extracted from cellular lysates by using RNA extraction kit (Sangon Biotech Co., Ltd., Shanghai, China) according to the manufacturer’s instructions. Briefly, genus (EC isolates) were inoculated into LB medium and incubated at 37°C with 250 rpm overnight. It was sub-cultured in LB medium with a concentration of 1:100. When absorbance of OD600 reached 0.8, bacteria were collected by centrifuge at 12,000*g* for 2 min and the supernatant was discarded, the precipitate was washed once with 1 ml of phosphate buffer (pH 7.0), bacterial pellet was resuspended in 100 μl of TE buffer containing 400 μg/ml lysozyme, and incubated for 5 min at room temperature. Next, 900 μl of lysis solution was added and mixed at room temperature for 3 min, 200 μl of chloroform (Sangon Biotech Co., Ltd., Shanghai, China) was added, mixed, and centrifuged at 12,000*g* at 4°C for 5 min. Consequently, 600 μl of supernatant (aqueous liquid) was acquired and 200 μl anhydrous ethanol was added, the mixture was incubated at room temperature for 3 min, centrifuged at 12,000*g* at 4°C for 5 min. The supernatant was discarded, and precipitate was washed with 70% ethanol twice, dried naturally, dissolved in ddH_2_O, the concentration of RNA was determined by NanoDrop^TM^8000 spectro-photometer (Thermo Fisher Scientific, Waltham, MA, United States) and stored at −70°C. For detecting the expression of AmpR target genes, LB medium containing 5 mg/L 1,6-anhydromuropeptides (anhMurNAc, Medicilon, Co., Ltd., Shanghai, China) was used at the stage of sub-culture.

### RT-qPCR Assays

cDNA was synthesized from 500 ng of total RNA with a FastKing gDNA Dispelling RT SuperMix kit (Tiangen Biotech Co., Ltd., Beijing, China) according to the manufacturer’s instructions. Real-time fluorescence quantitative PCR (qPCR) was performed with a SuperReal PreMix Color (SYBR Green) kit (Tiangen Biotech Co., Ltd., Beijing, China) according to the manufacturer’s instructions (volume: 20 μL. PCR program: pre-denaturation: 95°C/10 min. Denaturation: 95°C/30 s, Annealing:58°C/30 s, Elongation:72°C/30 s, and 30 cycles), with 16S as an internal control. Sequences of primers used in RT-qPCR assays are listed in the Supplementary Table 4. Each assay was performed independently at least three times.

### Protein Extraction and Western Blot Analysis

Total protein was extracted from EC by a bacterial protein extraction kit (Sangon Biotech Co., Ltd., Shanghai, China) according to the manufacturer’s instructions. Briefly, strains were inoculated into LB medium and incubated at 37°C with 250 rpm overnight. It was sub-cultured in LB medium with a dilution concentration of 1:100 and continue incubated at 37°C with 250 rpm. When absorbance of OD600 reached 0.8, bacteria were collected by centrifuge at 12,000*g* for 2 min and the supernatant was discarded, the precipitate was washed once with 1 ml of phosphate buffer (pH 7.0), bacterial pellet was resuspended 1 ml of protein lysate (Sangon Biotech Co., Ltd., Shanghai, China). Samples were placed on ice and lysed by sonication with a microprobe by using a 10-s pulse three times with a 10-s interval during each pulse. The samples were centrifuged at 10,000*g* for 10 min and supernatant was collected. The concentration of protein in supernatant was determined by a protein quantitative kit (Beyotime, Biotechnology, Shanghai, China), and 30 μg total protein was used to western blot assay. Western blot analysis was performed with a standard method as previously described (Yang et al., 2017). Information of antibodies used are as followings: rabbit anti-AmpC (Abnova Taipei, Taiwan, China), mouse anti-DnaK (Abcam, Cambridge, MA, United States), rabbit anti-NagZ (preparation by ourselves), goat anti-rabbit IgG-HRP (Santa Cruz Biotechnology, Inc., Santa Cruz, CA, United States), goat anti-mouse IgG-HRP (Santa Cruz Biotechnology, Inc., Santa Cruz, CA, United States). Images were taken with a SPOT-CCD camera. For quantitative analysis of western blot, intensities of protein bands were quantified by application of ImageJ, and DnaK was applied as an internal control. Each assay was performed independently at least three times.

### Construction of *nagZ*-Knockout EC Model

*nagZ*-knockout EC was obtained by homologous recombination method with application of a suicide vector (Luo et al., 2015). Briefly, two homologous arms of DNA fragments (A: 522 bp-upstream fragment of initiator codon, and B: 544 bp-downstream fragment of termination codon) of *nagZ* gene were obtained by PCR. The fusion DNA fragment (AB fragment: 1066 bp) was obtained by the fusion PCR. The fused DNA fragment of AB was cloned into the suicide plasmid pLP12 and verified by PCR and sequencing. The recombinant plasmid was transformed into *E. coli*β2163. Finally, *nagZ*-knockout *EC* strain was obtained by co-culture *E. coli*β2163 with DNA fragment AB and wild-type *E, cloacae*. The strains and reagents used in this experiment were purchased from Nuojing Biological Company (Knogen Biotech Co., Ltd., Guangzhou, China).

### Preparation of EC Models of NagZ Complementation

The CDS of *nagZ* was obtained by PCR, then cloned into a plasmid of pBAD33cm-rp4 (Knogen Biotech Co., Ltd., Guangzhou, China), and verified by sequencing. The recombinant plasmid (pBAD33-*nagZ*) was transformed into competent *E. coli*β2163 (Knogen Biotech Co., Ltd., Guangzhou, China). Finally, the recombinant plasmid from *E. coli* β2163 was transformed into *E. cloacae* by a conjugation assay, 0.05% L-Arabinose (Sangon Biotech Co., Ltd., Shanghai, China) was used to induce gene expressions of the recombinant plasmids. For antibiotic susceptibility test, L-Arabinose was added at the initial stage of antibiotic susceptibility test. For western blot, RNA Extraction and AmpC β-lactamase Activity Assay, L-Arabinose was added at the stage of sub-culture. Primers for obtaining CDS of *nagZ* are listed in the Supplementary Table 4.

### Statistical Analysis

All data were presented as mean ± standard deviation. Two-tailed *t*-test was used to determine the significant difference between two groups by GraphPad Prism 5. ^∗^*P* < 0.05 and ^∗∗^*P* < 0.01 were applied to be statistically significant and statistically highly significant, respectively. All experiments were performed independently at least three times.

## Results

### Enhanced NagZ and AmpC Expression in the Resistant EC Clinical Isolates

**FIGURE 1:**
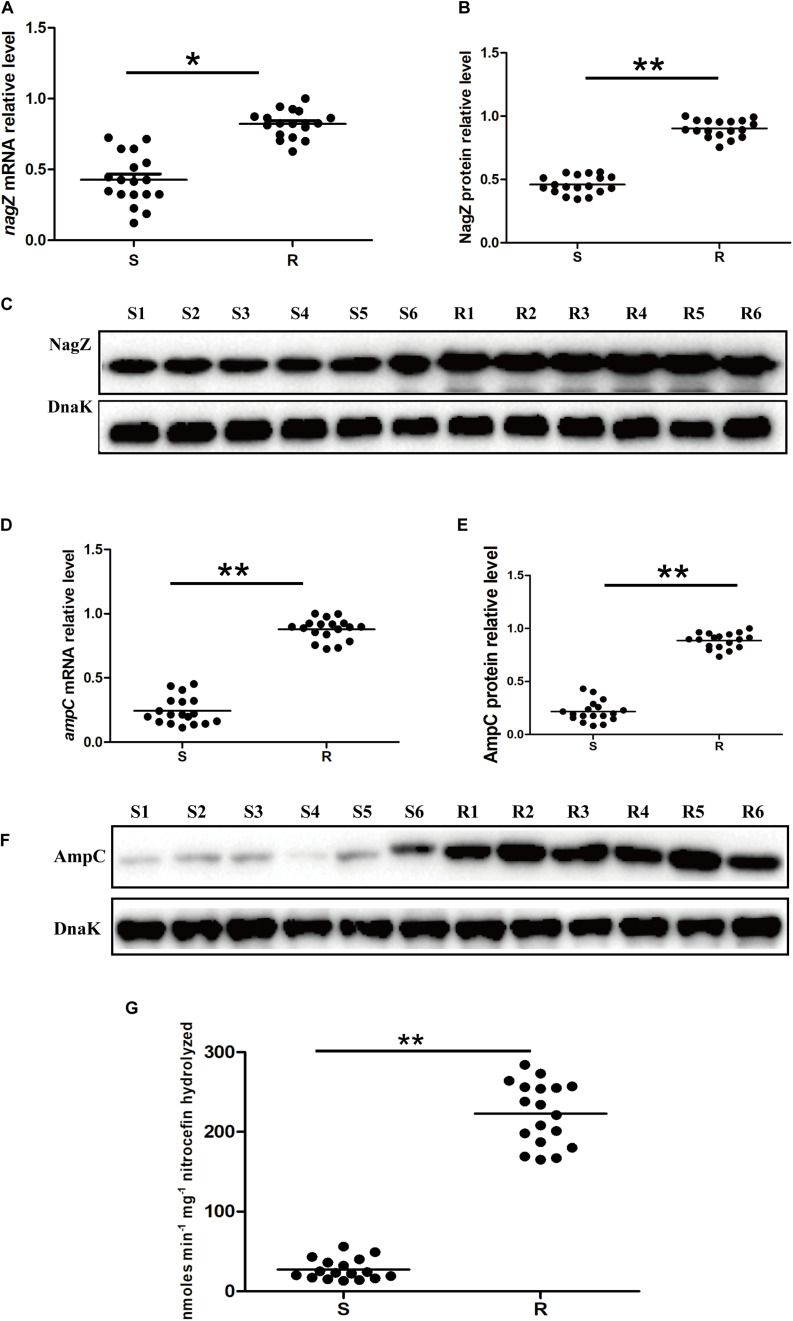
The expression levels of *nagZ* and *ampC* in *Enterobacter cloacae* (EC) isolated from clinical samples. **(A)** Quantification by reverse transcription-quantitative polymerase chain reaction (RT-qPCR) analysis of *nagZ* in susceptible and resistant EC isolates. **(B)** Quantitative analysis of the results of western blot **(C)**, DnaK was used as an internal control. **(C)** Western blot analysis of *nagZ* protein expression in the susceptible and resistant EC. **(D)** RT-qPCR analysis of *ampC* at mRNA level in susceptible and resistant EC strains. **(E)** Quantitative analysis of the results of western blot **(F)**, DnaK was used as an internal control. **(F)** Western blot analysis of *ampC* at protein level in susceptible and resistant isolates of EC. **(G)** AmpC β-lactamase activity (measured in nanomoles per minute per milligram nitrocefin hydrolyzed) were measured by nitrocefin hydrolysis assay. S, susceptible EC isolated from the clinical sample; R, resistant EC isolated from the clinical sample; S1, susceptible *EC* isolates number 1; R1, resistant *EC* isolates number 1, and so on. *P < 0.05 and **P < 0.01 indicate statistically significant and statistically highly significant, respectively.

To clarify mechanism of developing resistance in EC, 12 clinically isolated EC were randomly collected. Minimum inhibitory concentrations (MICs) of piperacillin (PIP), piperacillin-tazobactam (TZP), aztreonam (ATM), ceftriaxone (CRO), cefotaxime (CTX), cefoperazone (CFP), ceftazidime (CAZ), cefepime (FEP), imipenem (IMP), meropenem (MEM), levofloxacin (LVX), ciprofloxacin (CIP), amikacin (AMK) and gentamicin (GEN) against the 12 clinically isolated EC were determined according to protocols recommended by Clinical Laboratory Standard Institute (Supplementary Table 1; CLSI, 2018). Based on the MICs, 12 clinical isolates were divided into two groups (six susceptible and six resistant isolates, abbreviated as S1, S2, S3, S4, S5, S6, and R1, R2, R3, R4, R5, R6, respectively. Susceptible isolate: susceptible to all types of the third and fourth generation cephalosporins; resistant isolate: resistant to at least one type of the third or fourth generation cephalosporins). To determine whether NagZ was involved in developing resistance in EC, *nagZ* mRNA expression was examined by reverse transcription-quantitative polymerase chain reaction (RT-qPCR), as indicated in Figure 1A. *nagZ* mRNA expression was significantly enhanced in the resistant isolates compared with susceptible ones. To further detect the different protein expressions of *nagZ* between susceptible and resistant strains, we prepared anti-NagZ antibody for the first time, and its specificity was verified by *nagZ*-knockout EC model (Supplementary Figure 1). *nagZ* protein expressions were detected by western blot in six resistant and six susceptible strains, as indicated in Figures 1B,C, protein expressions of *nagZ* were dramatically up-regulated in six resistant EC isolates compared to susceptible ones.

NagZ is a cytosolic glucosaminidase and acts a crucial role in peptidoglycan recycling pathway, some publications reported there also exists a correlation between peptidoglycan recycling and *ampC* expression in *P. aeruginosa* (Reith and Mayer, 2011; Mayer, 2019). Therefore, mRNA expression level of *ampC* in 12 clinically isolated EC was determined by RT-qPCR (Figure 1D), the results indicated *ampC* mRNA expression was up-regulated in the resistant EC compared to the susceptible ones. Furthermore, protein expressions of *ampC* were enhanced in resistant EC isolates, which is shown in Figures 1E,F. Additionally, to investigate whether a highly expressed AmpC β-lactamase was associated with a higher β-lactamase activity, nitrocefin hydrolysis assay was used to determine the β-lactamase activity of AmpC. Our results confirmed that increasing protein level of AmpC had an excellent ability to hydrolyze nitrocefin (Figure 1G). All these data confirmed that expression of NagZ and β-lactamase activity of AmpC were enhanced in resistant EC isolates.

### NagZ Enhances Resistance to β-Lactam Antibiotics and Promotes AmpC Expression in Susceptible EC Isolates

**FIGURE 2:**
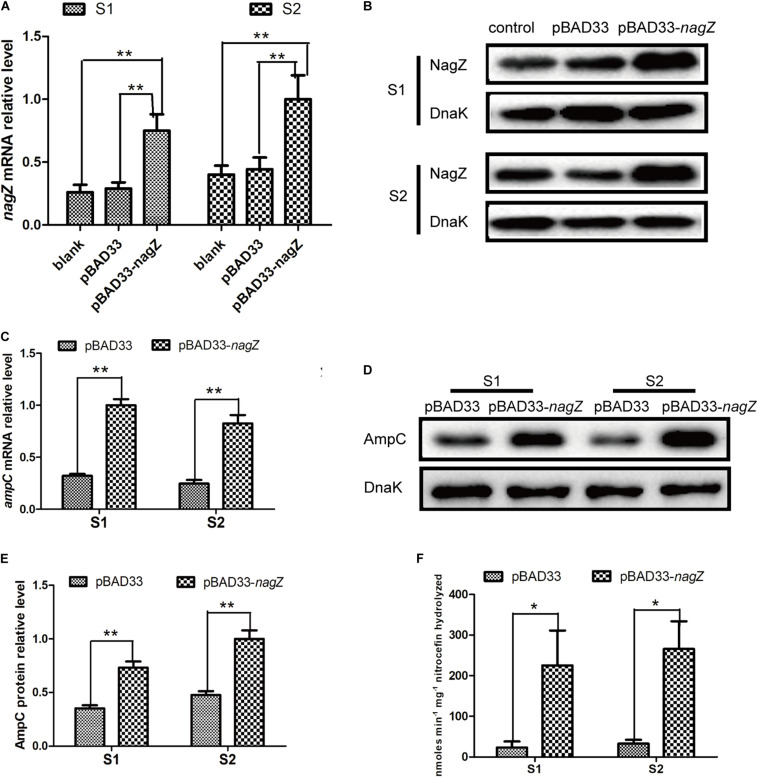
The effects of NagZ on expression of *ampC* in susceptible EC isolates. RT-qPCR **(A)** and western blot **(B)** confirmed that NagZ complementation vector (pBAD33-*nagZ*) was successful constructed. **(C)** Effects of NagZ complementation on mRNA expression of *ampC* in S1 and S2 isolates. **(D)** Western blot indicated effects of NagZ on AmpC β-lactamase in S1 and S2 isolates. **(E)** Quantitative analysis of western blot **(D)**, DnaK was used as an internal control. **(F)** Nitrocefin hydrolysis assay was used to evaluate role of NagZ in AmpC β-lactamase activity. pBAD33-*nagZ* and pBAD33 were used as the experiment group and control, respectively. *P < 0.05 and **P < 0.01 indicate statistically significant and statistically highly significant, respectively.

As indicated in Figure 1, our results demonstrated that *nagZ* expression was up-regulated in resistant EC isolates. It was further determined whether increased NagZ was significantly functional in developing resistance in EC. *nagZ* CDS was cloned into the pBAD33cm-rp4 vector (pBAD33-*nagZ*), and then pBAD33-*nagZ* (NagZ complementation vector) and a pBAD33cm-rp4 vector (pBAD33, control vector) were transformed into S1 and S2, respectively. RT-qPCR and western blot were used to detect whether pBAD33-*nagZ* vector was effective (Figures 2A,B), the results indicated that mRNA and protein expressions of *nagZ* were significantly increased complemented with the pBAD33-*nagZ* vector compared with pBAD33 vector. To further identify the role of NagZ in developing resistance, inhibition zones and MICs of PIP, TZP, ATM, CRO, cefoperazone-sulbactam (SCF), CAZ against S1 and S2 complemented with or without pBAD33-*nagZ* vector were determined by Kirby-Bauer method and broth microdilution according to Clinical Laboratory Standard Institute guideline (CLSI, 2018). As shown in Supplementary Figure 2A, inhibition zones of S1 and S2 complemented with pBAD33-*nagZ* were severely reduced compared with pBAD33. Furthermore, MICs of PIP, TZP, ATM, CRO, SCF, and CAZ were significantly increased in the EC complemented with pBAD33-*nagZ* compared to EC complemented with pBAD33 (Table 1). These results indicated that increased expression of NagZ enhanced resistance of EC to β-lactam antibiotics.

**TABLE 1:** The effect of NagZ on resistance in EC.

**Strain**	**MICs (μg/ml)**					
	**TZP**	**ATM**	**PIP**	**CRO**	**CAZ**	**SCF**
S1+pBAD33	2	1	2	0.125	0.25	0.5
S1+pBAD33-*nagZ*	8	128	16	128	256	8
S2+pBAD33	2	4	4	0.5	0.125	0.5
S2+pBAD33-*nagZ*	32	>512	64	256	512	2
S3+pBAD33	2	2	8	0.25	1	0.5
S3+pBAD33-*nagZ*	16	128	128	64	256	4
S4+pBAD33	4	1	8	0.5	0.5	0.5
S4+pBAD33-*nagZ*	32	64	64	64	256	8
S5+pBAD33	1	0.5	1	0.5	2	1
S5+pBAD33-*nagZ*	32	64	32	32	512	8
S6+pBAD33	0.25	1	0.5	1	0.25	1
S6+pBAD33-*nagZ*	16	256	32	128	64	8
R1	2	64	2	128	128	4
R-Δ*nagZ*	0.5	0.125	0.5	0.125	0.25	0.25
R1-Δ*nagZ*+pBAD33	0.5	0.125	0.5	0.25	0.5	0.25
R1-Δ*nagZ*+pBAD33-*nagZ*	8	64	8	64	64	8

*EC, *Enterobacter cloacae*; MIC, minimum inhibitory concentration; PIP, piperacillin; TZP, piperacillin-tazobactam; ATM, aztreonam; CRO, ceftriaxone; CAZ, ceftazidime; SCF, cefoperazone-sulbactam; S, susceptible EC isolated from the clinical sample; S1, susceptible EC isolates number 1, and so on; R1, resistant *Enterobacter cloacae* clinical isolates number 1; R1-Δ*nagZ*, *nagZ* knockout R1; pBAD33, control vector; pBAD33-*nagZ*, NagZ complementation vector; R1-Δ*nagZ*+pBAD33, R1-Δ*nagZ* complemented with pBAD33; R1-Δ*nagZ*+pBAD33-*nagZ*, R1-Δ*nagZ* complemented with pBAD33-*nagZ*.*

Next, we aimed to investigate whether expression of *ampC* is regulated by NagZ in EC isolates. pBAD33-*nagZ* and pBAD33 were transformed into S1 and S2, respectively. RT-qPCR and western blot were adopted to detect the effect of NagZ on AmpC expression. It is shown in Figures 2C–E, NagZ promoted mRNA (Figure 2C) and protein (Figures 2D,E) expressions of *ampC*. Nitrocefin hydrolysis assay was used to determine the β-lactamase activity of AmpC, results showed that AmpC hydrolysis activity was significantly improved in EC complemented with pBAD33-*nagZ* compared with pBAD33 (Figure 2F). Therefore, NagZ enhanced AmpC expression and increased AmpC β-lactamase activity in susceptible EC isolates.

### Knockout of *nagZ* Attenuated *ampC* Expression and Resistance to β-Lactam Antibiotics in EC Isolate

**FIGURE 3:**
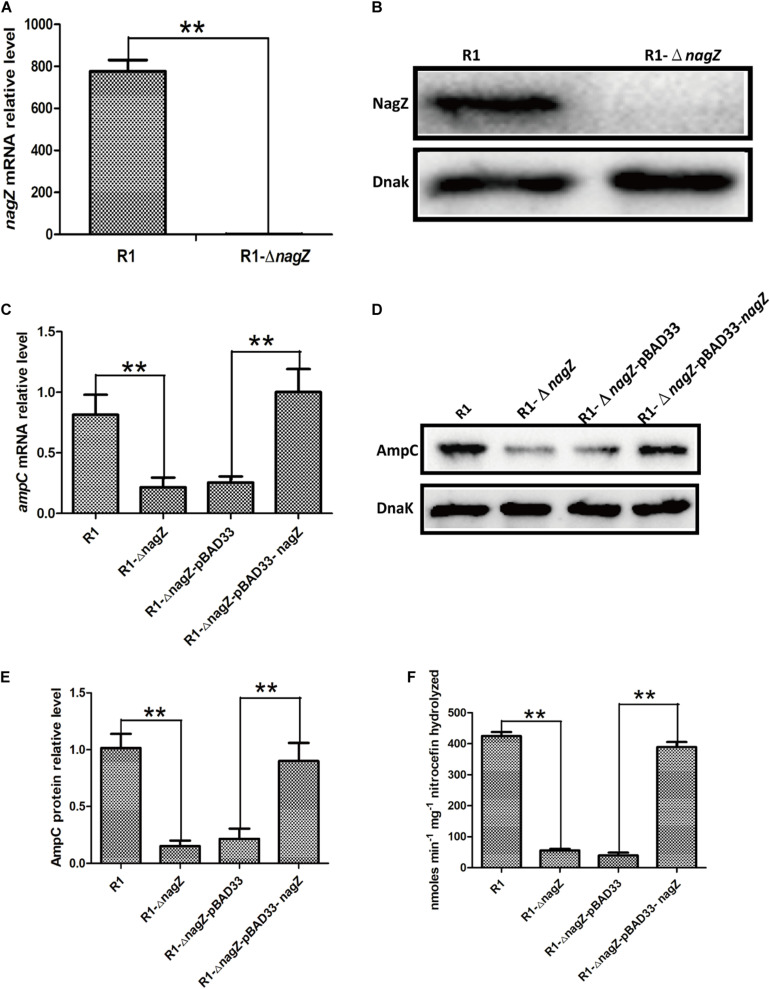
The effects of *nagZ* knockout and complementation on *ampC* expression. RT-qPCR **(A)** and western blot **(B)** confirmed that R1 *nagZ*-knockout model (R1-Δ*nagZ*) was successful prepared. **(C)** mRNA expressions of *ampC* were detected by RT-qPCR in strains of R1, R1-Δ*nagZ*, R1-Δ*nagZ-*pBAD33 (complemented with pBAD33 vector), and R1-Δ*nagZ-*pBAD33-*nagZ* (complemented with NagZ complementation vector). **(D)** Western blot was used to determine *ampC* protein expressions in R1, R1-Δ*nagZ*, R1-Δ*nagZ-*pBAD33, and R1-Δ*nagZ-*pBAD33-*nagZ*. **(E)** Quantitative analysis of the results of western blot **(D)**, DnaK was used as an internal control. **(F)** AmpC β-lactamase activity was analyzed by nitrocefin hydrolysis assay in R1, R1-Δ*nagZ*, R1-Δ*nagZ-*pBAD33, and R1-Δ*nagZ-*pBAD33-*nagZ*. **P < 0.01 indicate statistically highly significant.

To investigate the regulating role of NagZ in resistance and AmpC β-lactamase expression, we constructed a R1 *nagZ*-knockout model (R1-Δ*nagZ*) by homologous recombination. RT-qPCR (Figure 3A) and western blot (Figure 3B) confirmed that *nagZ* gene was successfully knocked out in clinical isolate of R1. Firstly, mRNA and protein levels of AmpC were detected by RT-qPCR and western blot, which suggested loss of NagZ reduced expression of *ampC* (Figures 3C–E). Secondly, nitrocefin hydrolysis assay indicated that β-lactamase activity of R1-Δ*nagZ* was significantly decreased compared with wild-type R1 (Figure 3F). Finally, the effect of NagZ on resistance of R1 was evaluated by broth microdilution and Kirby-Bauer method. The results suggested deletion of NagZ increased inhibition zones of CRO, CAZ, ATM, SCF, PIP, and TZP in R1 (Supplementary Figure 2B), while MICs of CRO, CAZ, ATM, SCF, PIP, and TZP against R1-Δ*nagZ* were at least fourfold lower than wild-type R1 (Table 1).

To further explore whether complementation of NagZ could rescue *ampC* expression and resistance in R1-Δ*nagZ* model, pBAD33 and pBAD33-*nagZ* were transformed into R1-Δ*nagZ* strain, respectively. RT-qPCR analyses confirmed that mRNA level of *ampC* was significantly increased by complementation of NagZ (Figure 3C). Moreover, the protein expression of *ampC* was rescued by NagZ complementation (Figures 3D,E). Furthermore, decreased β- lactamase activity induced by deletion of *nagZ* was rescued by complementation of NagZ (Figure 3F). In addition, inhibition zones and MICs of CRO, CAZ, ATM, SCF, PIP, and TZP against R1-Δ*nagZ* complemented with pBAD33 or pBAD33-*nagZ* vector were measured, and the results showed that increased inhibition zones induced by knockout of *nagZ* were reversed by complementation of NagZ (Supplementary Figure 2C). Consistently, knockout of *nagZ* significantly reduced MICs, which was rescued by complementation of NagZ as well (Table 1). In summary, NagZ promoted expression of *ampC* and β-lactamase activity, and enhanced resistance in strain of R1-Δ*nagZ*.

### NagZ Activates AmpR Through anhMurNAc

**FIGURE 4:**
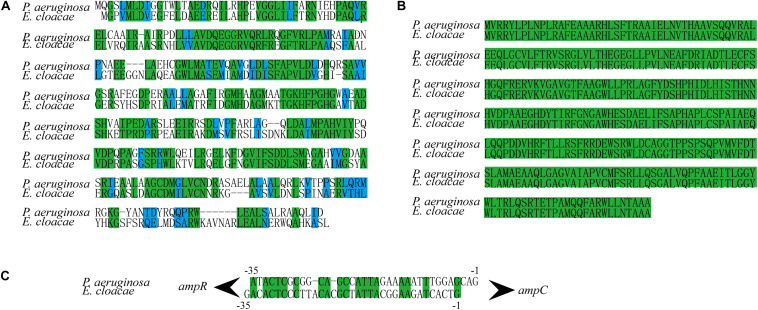
NagZ and AmpR sequence homology analysis between *Pseudomonas aeruginosa* and *Enterobacter cloacae*. **(A)** NagZ amino acid sequence alignment. **(B)** AmpR amino acid sequence alignment. **(C)** Upstream nucleotide sequence alignment of AmpC (about –35 bp). The identical sequences are marked by green, and those in blue belong to the same class of amino acids in terms of structure or function.

**FIGURE 5:**
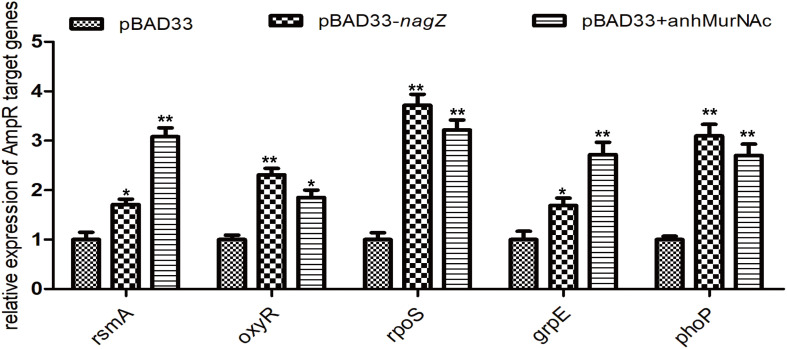
Effects of NagZ and anhMurNAc on expressions of AmpR target genes. *rsmA*, *oxyR*, *rpoS*, *grpE*, and *phoP* are target genes of AmpR. pBAD33, control vector; pBAD33-*nagZ*, NagZ complementation vector; anhMurNAc, the hydrolyzate of NagZ. *P < 0.05 and **P < 0.01 indicate statistically significant and statistically highly significant, respectively.

In *P. aeruginosa*, overproduction of the chromosomally encoded AmpC β-lactamase is the major mechanism of β-lactam resistance (Lodge et al., 1990; Kong et al., 2005). During normal physiological growth, *N*-acetylglucosaminyl-1,6-anhydromuropeptides (GlcNAc-1,6-anhydroMurNAc) are been transport into the cytoplasm by permease AmpG (Park and Uehara, 2008), where the glucosaminidase NagZ removes the GlcNAc moiety and form 1,6-anhydromuropeptides (anhMurNAc) (Park and Uehara, 2008; Ho et al., 2018). It has been proposed that anhMurNAc induces a conformational change of AmpR and maintains AmpR in an active conformation that promote the expression of *ampC* in *P. aeruginosa* (Caille et al., 2014), AmpR is a global transcriptional factor that regulates expression of hundreds of genes (such as *rsmA*, *oxyR*, *rpoS*, *grpE*, and *phoP*) (Kong et al., 2005; Caille et al., 2014). To explore the detail mechanism of NagZ promoting AmpC expression in *E. cloacae*, NagZ and AmpR Sequence homology were analyzed between *P. aeruginosa* and *E. cloacae*, the results revealed that *E. cloacae* NagZ (66.9%) and AmpR (100%) bears a high degree of homology to its counterpart *P. aeruginosa* (Figures 4A,B). A high degree of homology is also seen in the upstream region (transcriptional factor binding zone) of *ampC* between *P. aeruginosa* and *E. cloacae* (Figure 4C). Here, to further verify whether the regulation of NagZ on AmpC is mediated by the activation of AmpR by anhMurNAc, we examined the effect of NagZ on the expression of target genes of AmpR. The results indicate that NagZ can promote the expression of AmpR target genes such as *rsmA*, *oxyR*, *rpoS*, *grpE*, *phoP*, etc. (Figure 5). To further verify that the activation of AmpR is initiated by the NagZ hydrolyzate anhMurNAc, the effect of anhMurNAc on expression of AmpR target genes were examined. The results showed, consistent with NagZ, anhMurNAc could enhance the expression of *rsmA*, *oxyR*, *rpoS*, *grpE*, and *phoP* genes (Figure 5).

## Discussion

*Enterobacter cloacae* is ubiquitous in nature, existing in both terrestrial and aquatic environments. It is a well-known nosocomial pathogen that can cause multiple infections, such as lower respiratory tract infection, bacteremia, endocarditis, osteomyelitis, and etc. (Mezzatesta et al., 2012; Guerin et al., 2015). EC has an inherent resistance to ampicillin, amoxicillin, the first and second generation cephalosporins, and cefoxitin due to production of chromosomal AmpC β-lactamase (Pechere, 1991; Ito et al., 2018). Current literatures indicate overexpression of *ampC*, destruction of membrane permeability, and acquisition of plasmid-encoded carbapenemase genes are main mechanisms of carbapenem-resistant strain of EC (Cao et al., 2017; Rees et al., 2018; Wu et al., 2018). Despite those prominent studies, specific molecular mechanisms of chromosome-encoded AmpC β-lactamase in EC remain largely unknown. Our study provided three novel findings implicating NagZ could enhance the resistance of EC to β-lactam antibiotics. Firstly, there existed a strong positive correlation between the expression of *nagZ* and the resistance to β-lactam antibiotics, the expression of *nagZ* was increased in resistant EC isolates, and ectopic expression of *nagZ* enhanced resistance to β-lactam antibiotics in susceptible EC. Secondly, expression of NagZ is positively correlated with expression of AmpC, in resistant EC isolates, *nagZ* and *ampC* expression levels were significantly elevated, and AmpC β-lactamase activity was remarkably enhanced, specific complementation of NagZ could promote expression of *ampC* and enhance resistance of EC to β-lactam antibiotics. Our third novel finding is that NagZ hydrolyzate anhMurNAc promote the expression of target genes of AmpR, which indicates that NagZ regulates the expression of AmpC through the activation of AmpR by anhMurNAc.

Cell-wall remodeling, known as peptidoglycan recycling, is tightly regulated to guarantee bacterial survival (Gisin et al., 2013; Borisova et al., 2016). Cell-wall fragments produced during remodeling are recycled and act as signaling messengers for bacterial communication (Reith and Mayer, 2011). Emerging evidence indicates that peptidoglycan recycling pathway is strongly associated with the development of resistance, especially to β-lactams (Borisova et al., 2016; Gil-Marques et al., 2018; Torrens et al., 2019). Several enzymes or metabolites produced in peptidoglycan recycling can regulate expressions of antibiotics-resistant genes (Gomez-Simmonds et al., 2018). GlcNAc-1,6-anhydromuropeptide, a product generated during degradation of peptidoglycan, is transported into cytoplasm through AmpG (a transmembrane protein with a permease activity that transports meuropeptide from periplasm to cytoplasm) and then is hydrolyzed to form 1,6-anhydromuropeptides, which promotes the expression of β-lactamase in *P. aeruginosa* (Zamorano et al., 2010a; Yang et al., 2014; Huang et al., 2015a). Besides, stem peptides of GlcNAc-1,6-anhydromuropeptide and 1,6-anhydromuropeptides can be removed by AmpD (N-acetylmuramyl-L-alanine amidase) and eventually recycled to yield UDP-MurNAc pentapeptide, which inhibits β-lactamase expression (Juan et al., 2006; Balasubramanian et al., 2015; Liu et al., 2016). Moreover, penicillin-binding proteins (PBPs) play a vital role in regulation of β-lactamase (Pfeifle et al., 2000). In *P. aeruginosa*, PBP4, PBP5, and PBP7 are involved in AmpC β-lactamase regulation, and PBP4 is the main inhibitor of expression of AmpC β-lactamase (Moya et al., 2009; Ropy et al., 2015).

In this study, we proved that resistance to β-lactam in clinically isolated EC was closely relevant to the expression of *nagZ*. NagZ, encoded by gene *nagZ*, is a glucosaminidase present in Gram-negative bacteria, and acts a critical role in peptidoglycan recycling pathway by removing N-acetyl-glucosamine (GlcNAc) from degraded peptidoglycan. Here, we found that *nagZ* expression was increased at RNA and protein levels in clinically isolated resistant EC compared to susceptible ones. To test whether resistance of EC was caused by increasing expression of NagZ, NagZ complementation vector was constructed and transformed into susceptible EC. Our results indicated that complementation and knockout of *nagZ* could increase and decrease resistance to β-lactams in EC, respectively. These findings highlighted that NagZ plays a dispensable role in developing resistance of EC.

Another novel finding in this study was that expression of NagZ was discovered to be positively correlated with expression of AmpC and the activity of β-lactamase, in the resistant strains of EC, *nagZ* and *ampC* expression levels were significantly elevated, and AmpC β-lactamase activity was enhanced.

*ampC* is usually found in the chromosomes of *Enterobacteriaceae* (such as *Enterobacteria*) and non-fermenting bacteria (such as *P. aeruginosa*) (Jacoby, 2009). Overexpression of *ampC* makes bacteria resistant to penicillin, cephalosporins, monobactams, and carbapenems (especially with deficiency of membrane porin) (Quale et al., 2006; Majewski et al., 2016). In *P. aeruginosa*, overexpression of chromosomal AmpC β-lactamase is the major mechanism related with cephalosporin resistance, and occurs during exposure to β-lactam antibiotics which leads to inactivation of *ampD* and *dacB* (gene regulating *ampC* expression) (Zamorano et al., 2010a; Perez-Gallego et al., 2016). Constitutive overexpression of chromosomal AmpC β-lactamase in Gram-negative bacteria can develop antibiotic resistance and lead to a limited choice of antibiotics, since excessive AmpC causes development of resistance to multiple β-lactam antibiotics, including the third and fourth generation cephalosporins and carbapenem (Quale et al., 2006; Majewski et al., 2016). However, underlying molecular mechanisms are still poorly understood regarding to AmpC β-lactamase, especially its relation to peptidoglycan recycling.

To investigate whether resistance was relevant with the expression of *ampC* in EC, expression of *ampC* and activity of β-lactamase were determined in resistant strains of EC. In consistent with our hypothesis, expression of *ampC* and activity of β-lactamase were significantly up-regulated compared with the susceptible strains. Furthermore, expression of *ampC* and ability to hydrolyze β-lactams were also enhanced with overexpression of *nagZ* in susceptible strains of EC. To further study the interaction between NagZ and AmpC, *nagZ*-knockout EC was constructed. It was found that loss of NagZ resulted downregulation of *ampC* and weakened ability to hydrolyze β-lactam antibiotics in EC.

In addition, we evaluated the effects of NagZ and anhMurNAc on the expression of AmpR target genes except AmpC, the results show that NagZ and anhMurNAc could promote the expression of many AmpR target genes, which confirmed that NagZ regulate AmpC through activating transcription factor AmpR by anhMurNAc.

In conclusion, in present study, antibody against NagZ was prepared for the first time, and *nagZ*-knockout and complementation models in EC were successfully constructed. This is the first study that we have read about the mechanism of NagZ regulating AmpC in *E. cloacae.* We confirmed that NagZ promotes AmpC β-lactamase expression through activating AmpR, and enhances resistance to β-lactam antibiotics in *E. cloacae*, which is essential for the identification of novel potential drug targets.

## Data Availability Statement

The datasets presented in this study can be found in online repositories. The names of the repository/repositories and accession number(s) can be found in the article/Supplementary Material.

## Ethics Statement

The animal study was reviewed and approved by the Ethics Committee of the Clinical Medical College and the First Affiliated Hospital of Chengdu Medical College.

## Author Contributions

XGY and YX conceived this study and wrote the manuscript. XY and JZ contributed to searching literatures and writing manuscript. YZ, FW, HD, JZ, and FN performed the experiments. XP, DW, and YF contributed to designing experiments and analyzing the data. QZ and TB were responsible for reading and reviewing manuscript. XGY was the responsible person funded in this project. All authors have read and approved the final manuscript.

## Conflict of Interest

The authors declare that the research was conducted in the absence of any commercial or financial relationships that could be construed as a potential conflict of interest.

## Supplementary Material

Supplementary Figure 1 Western blot was used to evaluate specificity of anti-NagZ antibody, the result demonstrated that the antibody had a specific binding site (41 kD) to the total protein of *Enterobacter cloacae*. R1, resistant strain of *Enterobacter cloacae* of number 1; R1-Δ*nagZ*, *nagZ*-knockout R1.

Supplementary Figure 2 Antibiotic susceptibility test (Kirby-Bauer method) was used to identify impacts of NagZ on resistance in clinical isolates. **(A)** The effects of NagZ complementation on resistance were determined in S1 and S2 isolates. **(B)** The roles of *nagZ* knockout in resistance were determined in R1 isolate. **(C)** The effects of NagZ complementation on resistance were determined in R1-Δ*nagZ*.
