# Supplementary figures and images for: Elevating NagZ Improves Resistance to β-Lactam Antibiotics via Promoting AmpC β-Lactamase in Enterobacter cloacae
Source: Front Microbiol. 2020 Nov 4;11:586729. doi: 10.3389/fmicb.2020.586729 (PMC7672007; doi:10.3389/fmicb.2020.586729)

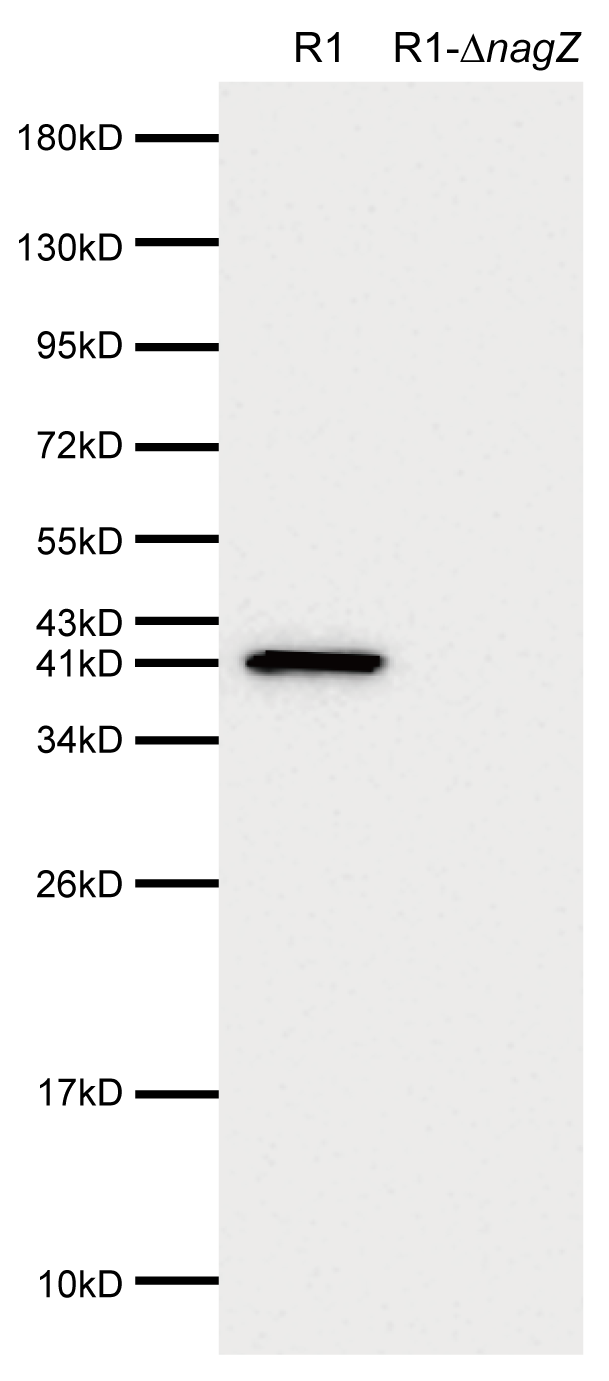

Supplement: Supplementary Figure 1 — Western blot was used to evaluate specificity of anti-NagZ antibody, the result demonstrated that the antibody had a specific binding site (41 kD) to the total protein of Enterobacter cloacae. R1, resistant strain of Enterobacter cloacae of number 1; R1-ΔnagZ, nagZ-knockout R1. [file Image_1.TIF]

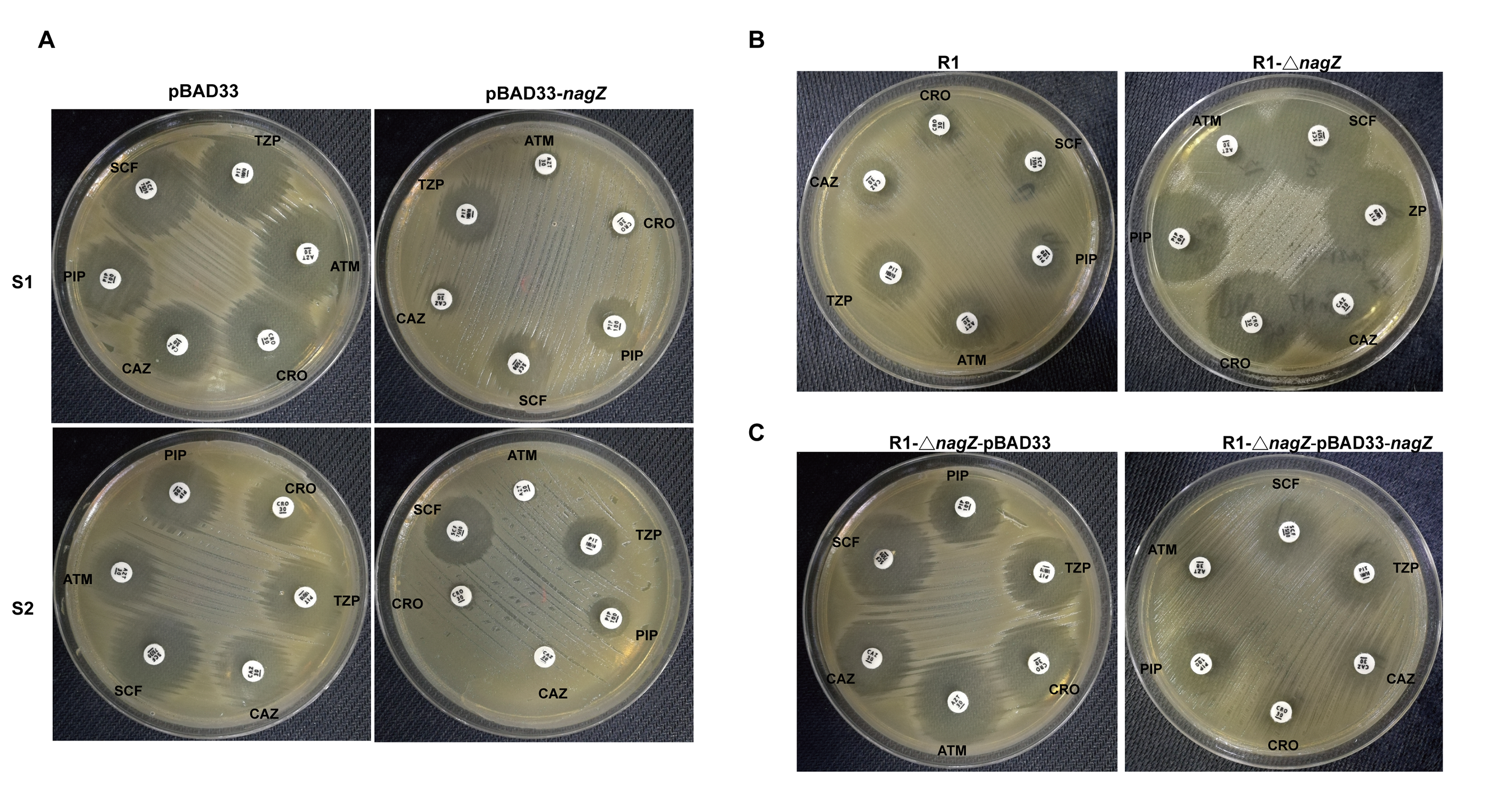

Supplement: Supplementary Figure 2 — Antibiotic susceptibility test (Kirby-Bauer method) was used to identify impacts of NagZ on resistance in clinical isolates. (A) The effects of NagZ complementation on resistance were determined in S1 and S2 isolates. (B) The roles of nagZ knockout in resistance were determined in R1 isolate. (C) The effects of NagZ complementation on resistance were determined in R1-ΔnagZ. [file Image_2.TIF]
