# Supplementary material for: Elevating NagZ Improves Resistance to β-Lactam Antibiotics via Promoting AmpC β-Lactamase in Enterobacter cloacae
Source: Front Microbiol. 2020 Nov 4;11:586729. doi: 10.3389/fmicb.2020.586729 (PMC7672007; doi:10.3389/fmicb.2020.586729)
Supplement: Supplementary file 3 [file Table_1.DOCX]

**TABLE S1 I** MICs of 14 antibiotics against 12 EC clinical isolates

| strains | sources | MICs (μg/ml） | | | | | | | | | | | | | |
| --- | --- | --- | --- | --- | --- | --- | --- | --- | --- | --- | --- | --- | --- | --- | --- |
|  |  | PIP | TZP | ATM | CRO | CTX | CFP | CAZ | FEP | IMP | MEM | LVX | CIP | AMK | GEN |
| S1 | blood | 2 | 2 | 1 | 0.125 | 0.25 | 2 | 0.25 | 0.5 | 0.25 | 0.125 | 0.125 | 0.25 | 2 | 0.5 |
| S2 | ascites | 4 | 2 | 4 | 0.5 | 0.5 | 2 | 0.25 | 0.25 | 0.25 | 0.25 | 0.25 | 0.5 | 1 | 0.5 |
| S3 | urine | 8 | 4 | 2 | 0.25 | 0.25 | 0.5 | 1 | 0.5 | 0.125 | 0.125 | 0.5 | 0.25 | 1 | 0.25 |
| S4 | urine | 4 | 4 | 1 | 0.5 | 0.25 | 1 | 0.5 | 0.25 | ＜0.125 | ＜0.125 | 0.25 | 0.5 | 2 | 0.5 |
| S5 | secretion | 1 | 1 | 0.5 | 0.5 | 0.5 | 0.5 | 1 | 0.5 | ＜0.125 | ＜0.125 | 0.125 | 0.125 | 0.5 | 0.5 |
| S6 | blood | 0.5 | 0.25 | 1 | 1 | 0.5 | 2 | 0.25 | 0.125 | 0.125 | ＜0.125 | 0.125 | 0.125 | 1 | 1 |
| R1 | blood | 4 | 2 | 64 | 256 | 128 | 256 | 64 | 8 | 0.5 | 0.25 | 16 | 8 | 256 | 32 |
| R2 | urine | 512 | 32 | 64 | 64 | 128 | 512 | 32 | 8 | 0.5 | 0.5 | 1 | 0.5 | 8 | 64 |
| R3 | sputum | 1024 | 32 | 64 | 256 | 256 | ＞1024 | 32 | 32 | 0.5 | 0.25 | 4 | 2 | 64 | 32 |
| R4 | sputum | 1024 | 16 | 512 | 512 | 256 | ＞1024 | 256 | 64 | 32 | 16 | 64 | 32 | 4 | 32 |
| R5 | hydrothorax | 1024 | 512 | 256 | 256 | 512 | 512 | 256 | 32 | 16 | 16 | 32 | 16 | 64 | 64 |
| R6 | urine | 512 | 16 | 32 | 128 | 64 | 1024 | 16 | 16 | 1 | 0.5 | 16 | 8 | 4 | 4 |

MIC: minimum inhibitory concentration. EC*: Enterobacter cloacae*, S1: susceptible EC clinical isolates number 1, R1: resistant EC clinical isolates number 2, and so on. PIP: piperacillin, TZP: piperacillin-tazobactam, ATM: aztreonam, CRO: ceftriaxone, CTX: cefotaxime, CFP: cefoperazone, CAZ: ceftazidime, FEP: cefepime, IMP: imipenem, MEM: meropenem, LVX: levofloxacin, CIP: ciprofloxacin, AMK: amikacin, GEN: gentamicin.
