# Supplementary material for: Elevating NagZ Improves Resistance to β-Lactam Antibiotics via Promoting AmpC β-Lactamase in Enterobacter cloacae
Source: Front Microbiol. 2020 Nov 4;11:586729. doi: 10.3389/fmicb.2020.586729 (PMC7672007; doi:10.3389/fmicb.2020.586729)
Supplement: Supplementary file 4 [file Table_2.DOCX]

**TABLE S2** **I** Strains information

| strains | sources | description |
| --- | --- | --- |
| EC-S1 | blood | antibiotic susceptibility tests, RT–qPCR, western blot, *nagZ* overexpression and AmpC β-lactamase activity assay. |
| EC-S2 | ascites | antibiotic susceptibility tests, RT–qPCR, western blot, *nagZ* overexpression and AmpC β-lactamase activity assay. |
| EC-S3 | urine | antibiotic susceptibility tests, RT–qPCR, western blot and AmpC β-lactamase activity assay. |
| EC-S4 | urine | antibiotic susceptibility tests, RT–qPCR, western blot and AmpC β-lactamase activity assay. |
| EC-S5 | secretion | antibiotic susceptibility tests, RT–qPCR, western blot and AmpC β-lactamase activity assay. |
| EC-S6 | blood | antibiotic susceptibility tests, RT–qPCR, western blot and AmpC β-lactamase activity assay. |
| EC-R1 | blood | Antibiotic susceptibility tests, RT–qPCR, western blot, *nagZ* overexpression, AmpC β-lactamase activity assay and gene *nagZ* knockout *EC* model construction. |
| EC-R2 | urine | antibiotic susceptibility tests, RT–qPCR AmpC β-lactamase activity assay, and western blot. |
| EC-R3 | sputum | antibiotic susceptibility tests, RT–qPCR, western blot, and AmpC β-lactamase activity assay. |
| EC-R4 | sputum | antibiotic susceptibility tests, RT–qPCR, western blot, AmpC β-lactamase activity assay. |
| EC-R5 | hydrothorax | antibiotic susceptibility tests, RT–qPCR, western blot, AmpC β-lactamase activity assay. |
| EC-R6 | urine | antibiotic susceptibility tests, RT–qPCR, western blot, AmpC β-lactamase activity assay. |
| EC ATCC 13047 | Bio-kont Co., Ltd | quality control of antibiotic susceptibility tests. |
| *Escherichia coli* ATCC 25922 | Bio-kont Co., Ltd | quality control of antibiotic susceptibility tests. |
| *Escherichia coli* DH5α | Knogen Biotech Co., Ltd | pET28a-*nagZ*-6His vector production. |
| *Escherichia coli B21* | Sangon Biotech Co., Ltd | NagZ-6His recombinant protein expression. |
| *Escherichia coli* β2163 | Knogen Biotech Co., Ltd | *nagZ* knockout construction and *nagZ* overexpression in EC*.* |

RT-qPCR*:* reverse transcription-quantitative polymerase chain reaction.
