# Supplementary material for: Elevating NagZ Improves Resistance to β-Lactam Antibiotics via Promoting AmpC β-Lactamase in Enterobacter cloacae
Source: Front Microbiol. 2020 Nov 4;11:586729. doi: 10.3389/fmicb.2020.586729 (PMC7672007; doi:10.3389/fmicb.2020.586729)
Supplement: Supplementary file 5 [file Table_3.DOCX]

**TABLE S3 I** Plasmid information

| plasmid | source | description |
| --- | --- | --- |
| pET28a | Sangon Biotech Co.,Ltd | construction of pET28a-*nagZ*-6His vector |
| pET28a-*nagZ*-6His | constructed in this study | *nagZ* overexpression in EC |
| pLP12 | Knogen Biotech Co., Ltd | *nagZ* knockout construction in EC |
| pBAD33cm-rp4(pBAD33) | Knogen Biotech Co., Ltd | *nagZ* knockout construction and *nagZ* overexpression in EC |
| pBAD33-*nagZ* | constructed in this study | *nagZ* overexpression in EC |
