# Supplementary material for: Elevating NagZ Improves Resistance to β-Lactam Antibiotics via Promoting AmpC β-Lactamase in Enterobacter cloacae
Source: Front Microbiol. 2020 Nov 4;11:586729. doi: 10.3389/fmicb.2020.586729 (PMC7672007; doi:10.3389/fmicb.2020.586729)
Supplement: Supplementary file 6 [file Table_4.DOCX]

**TABLE S4 I** Primers information

| Name | Primer sequences | product size(bp) |
| --- | --- | --- |
| *nagZ*-CDS Sense | 5' ATGTTGGATGTCGAAGGGTTTG 3' | 1014 |
| *nagZ*-CDS antisense | 5' TTAAAGGGCTGCTTTATGTGCC 3' |  |
| *ampC*-qPCR sense | 5' CGGATGAGGTCACGGATAAC 3' | 96 |
| *ampC*-qPCR antisense | 5' TGGCGTTGGCGTAAAGA 3' |  |
| *nagZ*-qPCR sense | 5' ATGCGGAGGAGCGTGAAAT 3' | 143 |
| *nagZ*-qPCR antisense | 5' GACGGCGACCACCAGATGA 3' |  |
| rsmA-qPCR sense | 5' AAAAGGCCAGGCGATGGTT 3' | 129 |
| *rsmA*-qPCR antisense | 5' GGGTGCGTTTGCAGACGAG 3' |  |
| *oxyR*-qPCR sense | 5' AAGCGGGAGCGGATGAAGATA 3' | 151 |
| *oxyR*-qPCR antisense | 5' ATGCACGGCAGGTAAACCACA 3' |  |
| *rpoS*-qPCR sense | 5' TACGATTCGCCTGCCGATTCAC 3' | 254 |
| rpoS-qPCR antisense | 5' CTTCCGGGCCGTTGTCTTTTTC 3' |  |
| grpE-qPCR sense | 5' GCGTCGTCGTACCGAACAGG 3' | 178 |
| *grpE*-qPCR antisense | 5' CAGCATGGATTTCAGCGTCA 3' |  |
| *phoP*-qPCR sense | 5' AGCCGATTATTATCTCAATGAACAC 3' | 132 |
| phoP-qPCR antisense | 5' GTCAGCACCAGGACAGGAAGG 3' |  |
| *ampD*-qPCR sense | 5' GTCAGCACCAGGACAGGAAGG 3' | 100 |
| *ampD*-qPCR antisense | 5' CTGCAATCGCAGGGTAAAGC 3' |  |
| 16S-qPCR sense | 5' TCCTACGGGAGGCAGCAGT 3' | 467 |
| 16S-qPCR antisense | 5' GGACTACCAGGGTATCTAATCCTGTT 3' |  |

CDS: coding sequence, qPCR: Real-time fluorescence quantitative polymerase chain reaction
